# Scene categorization by Hessian-regularized active perceptual feature selection

**DOI:** 10.1038/s41598-024-84181-x

**Published:** 2025-01-04

**Authors:** Junwu Zhou, Fuji Ren

**Affiliations:** 1 School of Higher Vocational and Technical College, Shanghai Dianji University, Shanghai, 201306 China; 2 College of Computer Sciences, Anhui University, Hefei, 230039 China

**Keywords:** Mathematics and computing, Computer science

## Abstract

Decoding the semantic categories of complex sceneries is fundamental to numerous artificial intelligence (AI) infrastructures. This work presents an advanced selection of multi-channel perceptual visual features for recognizing scenic images with elaborate spatial structures, focusing on developing a deep hierarchical model dedicated to learning human gaze behavior. Utilizing the BING objectness measure, we efficiently localize objects or their details across varying scales within scenes. To emulate humans observing semantically or visually significant areas within scenes, we propose a robust deep active learning (RDAL) strategy. This strategy progressively generates gaze shifting paths (GSP) and calculates deep GSP representations within a unified architecture. A notable advantage of RDAL is the robustness to label noise, which is implemented by a carefully-designed sparse penalty term. This mechanism ensures that irrelevant or misleading deep GSP features are intelligently discarded. Afterward, a novel Hessian-regularized Feature Selector (HFS) is proposed to select high-quality features from the deep GSP features, wherein (i) the spatial composition of scenic patches can be optimally maintained, and (ii) a linear SVM is learned simultaneously. Empirical evaluations across six standard scenic datasets demonstrated our method’s superior performance, highlighting its exceptional ability to differentiate various sophisticated scenery categories.

## Introduction

Correctly identifying the different types of scenes is essential for many AI systems^1–7^. For example, in smart navigation, knowing the best route from one place to another is crucial. This requires picking specific features like road layout, street directions, and city landscapes to improve navigation algorithms. In public safety, identifying features like road signs and changes in elevation helps enhance real-time monitoring of people and vehicles. Most vehicle accidents happen at intersections rather than on straight roads. By quickly and accurately classifying different scenes, we can use multi-camera systems at key intersections to watch for unusual activities involving cars and pedestrians.

**Fig. 1:**
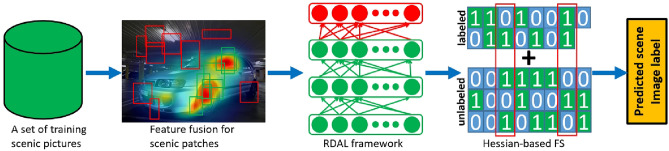
The pipeline of our designed hessian-regularized perceptual feature selection for scenery categorization.

In the field of visual categorization and labeling, various algorithms have been created to identify features in scenic images at different resolutions. Some popular methods include: (1) Multi-Instance Learning (MIL) and CNN-based strategies for object localization with minimal labeling^8,9^; (2) the use of semantic diffusion through graphical models for detailed interpretation of scenic images^10,11^; and (3) complex deep learning systems designed for comprehensive semantic annotation of scenic photos^12–14^.

However, these methods often struggle to accurately capture key scenic elements due to several challenges:

- High-resolution scenes have many important objects or components, requiring algorithms that mimic human perception of visually significant areas. Creating a deep learning framework to identify and refine these regions involves calculating the gaze shifting path (GSP) as humans focus on important image patches, handling noisy labels in large datasets, and embedding semantic labels at the patch level within each scene.
- Important regions in scenes are usually characterized by low-level descriptors that capture unique aspects of the scenery. Selecting these features effectively requires a method to weigh each feature channel appropriately, which is complicated by the need to adjust weights for different scenic datasets.

To address these challenges, we introduce a new scenery categorization method that uses deep active learning to model human gaze behavior, as shown in Fig. 1. Our approach begins with the BING objectness measure^15^ to extract object-aware patches from a collection of scenic images. Given a scene image, the BING objectness measure can rapidly and accurately generate a pre-specified number of object patches, each highly probably contains an object. We then use a low-level feature selection algorithm to encode the distribution of these patches. Our robust deep active learning (RDAL) framework calculates GSPs and their deep representations, effectively handling label noise and redundancy in a semi-supervised learning context. These deep GSP features are used in a multi-label SVM for diverse scenery classification. Tests on six public scenery datasets and a self-compiled sports image set demonstrated our method’s effectiveness.

The key innovations of this work are: 1. The RDAL framework, which actively learns human gaze behavior and calculates gaze-guided visual features. 2. A feature selection technique that dynamically calculates the importance of different feature channels for the deep GSP features.

## Review of prior work

In computer vision, many deep learning models for scene categorization use hierarchical Convolutional Neural Networks (CNNs) and complex architectures. These models work well with large image datasets like ImageNet, achieving high accuracy in scene categorization by using subsets of this dataset^16^. Despite being designed for general purposes, ImageNet-CNNs have proven useful in various computer vision tasks, such as video analysis and anomaly detection. Over the past decade, improvements to ImageNet-based CNNs have focused on two main areas: expanding training datasets and refining model architectures. Techniques like selective search have helped generate a wide range of patch samples by combining search strategies with semantic annotations^17^. Region-level CNNs (R-CNNs) have aimed at extracting high-quality patch samples for detailed image analysis^18^. Other advancements include creating large-scale, scenery-specific datasets for training^19^ and using pre-trained hierarchical CNNs to identify and represent local scenic patches^20^. Multi-task and multi-resolution algorithms for scenery classification have also been developed, using manifold-based regularization to maintain feature distributions^21^ and low-rank feature learning combined with Markov models for semantic annotation and contextual understanding^22^. Unsupervised learning models have been explored for deep feature extraction from scenic images, using geometric features to learn without labeled data^23^. Additionally, integrated models that combine discriminative feature learning with weak label learning have been proposed, using stacked sparse autoencoders for high-level visual representation^24^.

Many computational models have been developed to analyze aerial images using advanced machine learning techniques. In^25^, a multi-modal learning framework was introduced to effectively annotate high-resolution (HR) aerial images. Similarly,^26^ explored a new multi-attention mechanism to evaluate the importance of features in aerial photographs. These approaches are useful for classifying aerial images at different resolutions but struggle with low-resolution (LR) images because small, important objects can appear blurry. To address this, region-level modeling is crucial for accurately detecting and locating these critical objects in LR aerial images. To improve facial recognition in such contexts,^27^ introduced a group sparsity regularizer that refines the $l_1$-norm to reduce bias and outlier effects. Additionally,^28^ addressed incomplete multi-view clustering by enhancing incomplete similarity graphs and learning comprehensive tensor representations. For detailed regional characterization of aerial images,^29^ proposed a multi-layer deep learning approach to identify objects of interest at various scales. A focal-loss-based deep learning model was created in^30^ to accurately pinpoint vehicle locations in both LR and HR aerial photos. Another advance was made by^31^, who developed a model for geographic object detection in HR images, focusing on extracting critical features like intersections and roadways. Lastly,^32^ combined feature engineering with soft-label techniques to create a powerful visual detection framework for aerial image analysis.

Recently,^1^ proposed a novel approach that incorporates clustering mechanisms into neural networks to improve visual representation learning. The method aims to enhance the quality and robustness of visual features extracted from images, addressing limitations in traditional visual representation techniques. In^2^, the authors presented a new method that merged unsupervised sub-pattern discovery with supervised representation learning through a distance/case-based classification approach. This method capitalized on the strengths of both learning paradigms to boost visual recognition performance. Wang et al.^3^ introduced a method to infer salient objects from human fixations, bridging computational saliency modeling and human perception. It provides valuable insights for applications like object detection and image analysis. Researchers^4^, proposed a deep learning approach for visual attention prediction, facilitating content-aware designs and automated surveillance. The authors^5^, presented a weakly supervised semantic segmentation framework leveraging multi-image contexts to improve segmentation accuracy, which is critical for domains like medical imaging. Wang et al.^6^ utilized super-trajectories for semi-supervised video object segmentation, effectively addressing challenges in video analytics and object tracking. Lastly, the authors^7^ developed a deep learning solution for attention and aesthetics-aware photo cropping, automating the process of selecting visually appealing compositions, with applications in photo editing tools.

## Our scenery understanding pipeline

### Object-aware patch extraction

Research in visual cognition and psychology^33,34^ has shown that humans tend to focus on important parts of a scene, whether they are semantically or visually significant. This selective attention highlights key areas for cognitive processing. Integrating this aspect of human gaze behavior into scene categorization algorithms is essential. We propose a method that uses efficient object-aware patch detection and a robust Deep Active Learning (RDAL) framework to mimic human visual perception by focusing on important scenic patches. Studies show that humans are naturally drawn to significant objects or their components, like vehicles and skyscrapers, influenced by their spatial arrangements within the scene. To capture such elements, we use the BING objectness measure^15^, known for its effectiveness in identifying high-quality, object-aware patches across different scenes. The BING algorithm has three main advantages: its efficiency in detecting relevant object patches with minimal computational effort, its ability to significantly enhance GSP extraction by generating a wide range of object-level patches, and its excellent adaptability to new object categories. These features ensure our scene categorization model is effective across varied datasets.

BING, which stands for Binarized Normed Gradients, is a method developed for quickly estimating objectness in images. Objectness estimation involves determining whether a region in an image contains an object or not. BING achieves this at an impressive speed of 300 fps. The method extracts gradient features from an image, which capture the edges and outlines of objects. These gradients are then normalized to ensure scale-invariance, and binarized, converting the gradient information into a binary form to significantly reduce computational complexity. This simplification enables BING to perform object detection very rapidly, making it ideal for applications such as autonomous driving, real-time surveillance, and interactive computer vision systems. Additionally, BING can serve as a fast preprocessing step to identify potential object regions for further analysis by more complex algorithms. The significance of BING lies in its innovative approach to objectness estimation. Traditional methods often struggle with balancing speed and accuracy, especially in real-time scenarios. BING overcomes these challenges by leveraging simple yet powerful gradient-based features and innovative normalization and binarization techniques. The binarization process, in particular, transforms the normalized gradients into a form that is computationally inexpensive to process, allowing the method to maintain high speed without sacrificing performance. This makes BING highly efficient and effective in identifying object regions within images, facilitating quicker and more accurate object detection. BING’s ability to process images at 300 fps ensures it can be integrated into various applications that require instantaneous feedback. In autonomous driving, for instance, the rapid detection of objects such as pedestrians, vehicles, and obstacles is crucial for safe navigation. Similarly, in real-time surveillance, quick identification of potential threats or anomalies can enhance security measures. Interactive computer vision systems, which are increasingly used in gaming and augmented reality, also benefit from BING’s fast processing capabilities.

### Robust deep active learning (RDAL) framework

We leverage the BING objectness measure^15^ to extract a set of scenic patches from each scenery. In detail, by setting *N* as the number of acquired object patches for each scenic picture, the BING objectness measure can efficiently and effectively output *N* image patches that are highly responsive to each internal object in the scenic picture. However, human visual attention typically focuses on only a few key objects in a scene. To model this selective perception, we introduce a new Robust Deep Active Learning (RDAL) strategy. This approach is designed to identify *L* key scenic patches for creating GSP, from which deep GSP representations are computed. The RDAL framework integrates three key aspects: the spatial layout of the scene, the semantic richness of object patches, and the handling of potentially incorrect semantic labels.

Spatial composition in scenic analysis: Accurately modeling the spatial arrangement of elements in a scene, separating foreground from background, is crucial for advanced scene categorization. This involves analyzing the layout of the scene, where each scenic patch is defined in relation to its neighboring patches. By quantifying these spatial relationships, we can assess the significance of each object patch within the overall scene. This is done through an optimization process that assigns weights to each patch based on their spatial connectivity and relevance, improving the model’s ability to understand and categorize complex scenes effectively.

1 $$\begin{aligned} & \arg \min _\mathbf{E}\sum \limits _{i=1}^{N} \left\| z_i-\sum \limits _{j=1}^{N}\mathbf{F}_{ij}z_j \right\|\nonumber \\ & s.t.~\sum \limits _{j=1}^N \mathbf{F}_{ij}=1,\mathbf{F}_{ij}=0~~if~~z_i\notin \mathscr {N}(z_j), \end{aligned}$$

Here, $\{z_1,\ldots ,z_N\}\in \mathbb {R}^{N\times A}$ represents deeply-learned features from the *N* scenic patches identified by BING^15^ in each scene. The dimension of each scenic patch’s deep representation is *A*, and the matrix $\mathbf{F}_{ij}$ indicates the importance of the *i*-th scenic patch in reconstructing the *j*-th scenic patch. Additionally, $\mathscr {N}(z_i)$ includes the patches that are spatially adjacent to the scenic patch $z_i$.

Semantic significance of scenic patches: Besides capturing the spatial layout of scenes, the semantic integrity of selected scenic patches is crucial for constructing GSPs. Using a defined reconstruction error, we represent the scenic patches as ${g_1,\ldots ,g_N}$. The selection of *L* semantically important patches is achieved by minimizing a specific formulation, ensuring that these patches accurately reflect the scene’s key semantic attributes necessary for effective GSP construction.

2 $$\begin{aligned} & \eta (g_1,\ldots ,g_N)\nonumber \\ & \quad =\sum \limits _{i=1}^{L}||g_{q_i}-g_{q_i}||^2+ \tau \sum \limits _{i=1}^{N}\left\| g_i-\sum \limits _{j=1}^{N}\mathbf{F}_{ij}g_j \right\|^2, \end{aligned}$$

Here, $\tau$ weights the regularizer, and ${g_{q_1},\ldots ,g_{q_K}}$ represents the set of *L* scenic patches selected by our RDAL method. The first term minimizes the cost to fix the coordinates of the chosen samples. The last term ensures that the semantically reconstructed scenic patches are very similar to the input. Overall, minimizing (2) results in a collection of scenic patches that accurately reflect how humans visually and semantically perceive different scenes.

We define matrices $\mathbf{A}=[z_1,\ldots ,z_N]$ and $\mathbf{H}=[g_1,\ldots ,g_N]$, and let $\pmb {\Delta }$ be an $N\times N$ diagonal matrix representing the selected scenic patches. In this context, $\pmb {\Delta }_{ii}=1$ if $i\in \{q_1,\ldots ,q_L\}$ and 0 otherwise. This allows us to upgrade the objective function (2) as follows:

3 $$\begin{aligned} \eta (\mathbf{Q})=\text {tr}((\mathbf{H}-\mathbf{A})^T\Delta (\mathbf{H}-\mathbf{A}))+\tau \text {tr}(\mathbf{H}^T\mathbf{LH}), \end{aligned}$$

Herein, we have $\mathbf{L}=(\mathbf{I}-\mathbf{F})^T(\mathbf{I}-\mathbf{F})$. For optimizing (9), we set $\eta (\mathbf{H})$’s gradient to zero, and thereby we have:

4 $$\begin{aligned} \pmb {\Delta }(\mathbf{H}-\mathbf{A})+\tau \mathbf{LH} = 0. \end{aligned}$$

In this context, we can compute the rebuilt scenic patches as follows:

5 $$\begin{aligned} \mathbf{H} = (\tau \mathbf{L}+\pmb {\Delta })^{-1}\pmb {\Delta A}. \end{aligned}$$

By leveraging the reconstructed scenic patches, we can upgrade the reconstruction error as:

6 $$\begin{aligned} \eta (z_{q_1},\ldots ,z_{q_K})= & ||\mathbf{A}-\mathbf{H}||_F^{2}=||\mathbf{A}-(\tau \mathbf{L}+\pmb {\Delta })^{-1}\pmb {\Delta A}||_F^{2}\nonumber \\= & ||(\tau \mathbf{L}+\pmb {\Delta })^{-1}\tau \mathbf{LA}||_F^{2}, \end{aligned}$$

Here, $||\cdot ||_F^{2}$ represents the Frobenius norm for a matrix.

**Fig. 2:**
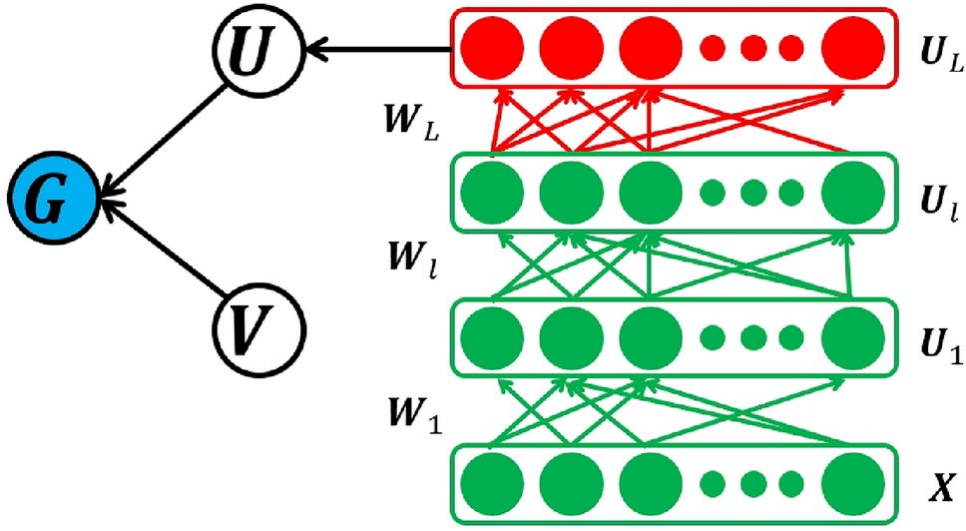
Structure of the designed deeply and semantically GSP encoding.

The RDAL framework: Our approach uses deep learning to hierarchically extract semantic visual descriptors from scenic images. As shown in Fig. 2, in an *R*-layer deep architecture, our Robust Deep Active Learning (RDAL) strategy involves breaking down the semantic label matrix $\mathbf{H}$ into $R+1$ distinct matrices: $\mathbf{V}$, $\mathbf{U}_R,\ldots ,\mathbf{U}_1$. This breakdown helps extract deep features relevant to each scene and efficiently represent new scenic images. Specifically, feature extraction at the first layer is performed using the equation $\mathbf{Q}_1=\mathbf{U}_1\mathbf{Y}$, where $\mathbf{U}_1$ is the weight matrix applied to the input features $\mathbf{Y}$. The RDAL framework is based on the principle of using linear combinations to uncover and refine hidden scenic attributes. This method creates a hierarchical, multi-layer architecture that provides a detailed understanding of scenic elements through successive layers of feature transformation and optimization.

7 $$\begin{aligned} & \mathbf{H}\leftarrow \mathbf{UQ}_R,\nonumber \\ & \mathbf{Q}_R=\mathbf{U}_R\mathbf{P}_{R-1},\nonumber \\ & ~~~\ldots \nonumber \\ & \mathbf{Q}_1=\mathbf{U}_1\mathbf{Y}, \end{aligned}$$

In this context, $\mathbf{U}_i$ is the transformation matrix for the *i*-th layer of the deep learning architecture, responsible for feature transformation. $\mathbf{P}$ represents the matrix of hidden semantic labels within the dataset. The scene representation matrix at the *i*-th layer, denoted as $\mathbf{Q}_i$, contains the aggregated scene features after transformation. Additionally, $\mathbf{Y}$ consists of vectors $y_i$, each a *B*-dimensional combination of features from the *i*-th scenic patch. In our Robust Deep Active Learning (RDAL) framework, the final layer’s deep representation is $\mathbf{Q}_R$. According to Eq. (7), training our deep model focuses on finding the matrix $\mathbf{P}$ and a series of *R* transformation matrices, $\mathbf{U}_R,\ldots ,\mathbf{U}_1$, to systematically learn and represent scenic elements at different network depths.

In summary, the entire deep-model-guided active learning can be mathematically represented as:

8 $$\begin{aligned} & \min _{\mathbf{P},\pmb {\Delta }\mathbf{U}_1,\ldots ,\mathbf{U}_R} \frac{1}{2}||\mathbf{K}-\mathbf{UP}||_F^2+\frac{\alpha }{2}||\mathbf{P}||_F^2+\frac{\alpha }{2}\sum \nolimits _{i=1}^R||\mathbf{U}_i||_F^2\nonumber \\ & \qquad +\frac{\beta }{2}||\mathbf{U}||_{2,1}, \end{aligned}$$

In this framework, the matrix $\mathbf{K} \in \mathbb {R}^{R\times N}$ contains semantic labels, where $\mathbf{K}_{ij}=1$ means the *i*-th scenic image is associated with the *j*-th label, and $\mathbf{K}_{ij}=0$ means it is not. Here, *R* represents the total number of unique semantic labels. The regularization parameters $\alpha$ and $\beta$ help prevent overfitting and enforce column-wise sparsity in the transformation matrix $\mathbf{U}_i$. Because visual features can be correlated, redundant, or noisy, we use an $l_{2,1}$-norm sparse regularization strategy to filter out low-quality features. Unlike traditional visual feature analysis (which looks at scene spatial composition and patch-level semantic details), our Robust Deep Active Learning (RDAL) model uses a semi-supervised learning approach. This method requires only a small set of semantic labels for training, as described in (8), making it useful when it is impractical to manually label large image datasets.

### Hessian-regularized feature selector

Let $\mathbf{C}=[c_1,\ldots ,c_N]\in \mathbb {R}^{N\times T}$ represent the matrix encapsulating deep GSP features derived from all training instances. For simplicity, we assume the initial *L* scenic images are annotated ($\mathbf{C}_L=[c_1,\ldots ,c_L]$), with the remainder being unlabeled ($\mathbf{C}_U=[c_{L+1},\ldots ,c_N]$). The label matrix for these *L* labeled instances is denoted as $\mathbf{M}=[m_1,\ldots ,m_L]$. The matrix $\mathbf{A}\in \mathbb {R}^{T\times C}$ is introduced to indicate the selection of features, where *C* represents the number of aerial image categories. The feature selection strategy is then articulated through the optimization of the following objective function, aiming to identify the most discriminative features for categorization tasks within this structured framework.

9 $$\begin{aligned} \min _{\mathbf{A}} \varepsilon (\mathbf{A})+\varphi \cdot \eta (\mathbf{A}), \end{aligned}$$

Here, $\varepsilon (\mathbf{A})$ is the loss function used to evaluate the performance of the selected features, and $\varphi \cdot \eta (\mathbf{A})$ is the regularization term, with $\varphi$ as the regularization coefficient balancing the loss function.

An affinity graph is denoted by $\mathbf{B}$, where each element $\mathbf{B}_{ij}$ measures the similarity between the *i*-th and *j*-th samples based on their proximity in feature space. Typically, $\mathbf{B}_{ij}=1$ indicates that samples *i* and *j* are neighbors, showing spatial or feature-space closeness, while $\mathbf{B}_{ij}=0$ means there is no direct affinity between them. Additionally, $\mathbf{D}$ is a diagonal matrix with elements $\mathbf{D}_{ii}=\sum _j \mathbf{B}_{ij}$, which helps compute the graph Laplacian $\mathbf{N}=\mathbf{D}-\mathbf{B}$. Preserving the structure of $\mathbf{N}$ during feature selection is crucial for maintaining the intrinsic geometric relationships among samples.

In this study, we analyze both labeled and unlabeled samples using a transductive learning approach^35^. We introduce the predicted label vector for scenic images as $\mathbf{J}=[j_1,j_2,\ldots ,j_N]^T \in \mathbb {R}^{N\times C}$, covering all training instances, where $j_i$ represents the inferred label for the *i*-th training sample. Consistent with the principles in^35^, we aim for $\mathbf{J}$ to align closely with both the ground-truth label matrix $\mathbf{M}$ and the affinity graph we construct, ensuring that $\mathbf{J}$ accurately reflects the true labels. The derivation of $\mathbf{J}$ is achieved by optimizing a well-defined objective function, effectively integrating transductive learning to bridge the gap between known and unknown sample labels.

10 $$\begin{aligned} \arg \min _{\mathbf{J}} \text {tr}(\mathbf{J}^T\mathbf{NJ})+\text {tr}((\mathbf{J}-\mathbf{M})^T\mathbf{O}(\mathbf{J}-\mathbf{M})), \end{aligned}$$

The matrix $\mathbf{O}$ is a diagonal matrix created based on a specific rule. If the *i*-th sample in the dataset is labeled, $\mathbf{O}_{ii}$ is set to a very large number (like 1*e*10). For unlabeled samples, $\mathbf{O}_{ii}$ is set to 1. This rule ensures that the predicted labels closely match the actual ground truth, making sure the calculated labels are as accurate as possible.

11 $$\begin{aligned} & \arg \min _{\mathbf{J},\mathbf{C}} \text {tr}(\mathbf{J}^T\mathbf{NJ})+\text {tr}((\mathbf{J}-\mathbf{M})^T\mathbf{O}(\mathbf{J}-\mathbf{M}))\nonumber \\ & \qquad +\beta ||\mathbf{C}^T\mathbf{A}||_F^2+\varphi \cdot \eta (\mathbf{A}), \end{aligned}$$

In this framework, $\beta$ is the coefficient for the regularization term. The objective function ensures that the predicted scenic image labels $\mathbf{J}$ closely match both the ground truth labels and the constructed affinity graph during the semi-supervised learning process. At the same time, the regularization term $\varphi \cdot \eta (\mathbf{A})$ enforces sparsity on the feature selection matrix $\mathbf{A}$. Additionally, the term $\beta ||\mathbf{C}^T\mathbf{A}||_F^2$ adds a penalty to improve label prediction accuracy. This setup allows for the simultaneous calculation of the linear classifier ($\mathbf{A}$) and the predicted labels ($\mathbf{J}$).

In machine learning, designing feature selectors often involves using various regularizers to optimally identify high-quality, discriminative features. The $l_{2,p}$-norm is commonly used to promote feature sparsity. Studies cited in^36^ show that the $l_{2,1/2}$-norm significantly improves the robustness and discriminative power of selected features. When $p=1/2$, this regularization strategy is called a Hessian regularizer. Therefore, the objective function is adjusted to include this Hessian regularization, optimizing feature selection in the semi-supervised learning context.

12 $$\begin{aligned} & \arg \min _{\mathbf{J},\mathbf{C}} \text {tr}(\mathbf{J}^T\mathbf{NJ})+\text {tr}((\mathbf{J}-\mathbf{M})^T\mathbf{o}(\mathbf{J}-\mathbf{M}))\nonumber \\ & \qquad +\beta ||\mathbf{C}^T\mathbf{A}||_F^2+\varphi \cdot ||\mathbf{A}||_{2,1/2}^{1/2}, \end{aligned}$$

Mathematically, optimizing the $l_{2,1/2}$-norm in the Hessian regularization framework is non-convex. To solve this, we use an iterative algorithm, as detailed in^37^.

## Empirical results

In this study, we thoroughly evaluate our scene classification framework, based on the RDAL approach, through four different experiments. First, we describe the experimental setups and introduce six benchmark datasets for scene analysis. Next, we compare our framework with various classifiers, including shallow learning models and deep multi-layer architectures. Then, we examine how key parameters in our RDAL framework affect classification results. Finally, we test the usefulness of the deep GSP features in improving categorization accuracy for educational sports images.

### Datasets and setting

Our categorization model is thoroughly tested on six different scenic image datasets, including both well-known benchmarks and newer collections. The main datasets in our study are Scene-15^38^ and MIT Indoor Scene-67.

We also test our pipeline on four modern scenic image collections: ZJU aerial imagery, ILSVRC-2010, SUN, and Places. Additionally, we introduce a new dataset, the Massive-Scale Sport Educational Imagery (MSEI), created specifically for educational purposes in sports. This large dataset contains about 920,000 images across nine sports categories, including basketball, football, volleyball, outdoor golf, athletics, table tennis, rowing, baseball, and equestrian. Detailed metrics for each dataset are provided in Table 1.

**Table 1:** Statistics of our compiled sport image set.

Sport name	Training image #	Testing image #
Basketball	83,132	23,467
Football	74,658	25,760
Volleyball	73,890	29,833
Golf	83,241	25,443
Athletics	65,457	34,556
Baseball	68,760	21,276
Tennis	73,879	25,465
Rowing	74,806	24,776
Equestrian	82,760	20,065

Before evaluating against baseline models, we outline the setup of our methodology: 1. Object Patches: Using the BING algorithm^15^, we extract 1000 scenic patches per dataset across all six collections. This standardization ensures thorough detection of potential objects within the scenes. 2. Spatial Neighbors: The number of spatial neighbors, denoted by *L*, is set to five. This reflects typical human visual attention, which focuses on a few key areas within a scene. 3. Low-level Features: For each object patch, we use three low-level features to capture essential visual details: a 16-dimensional color moment descriptor^39^, a 64-dimensional Histogram of Oriented Gradients (HOG)^40^, and a 160-dimensional edge and color histogram^41^. 4. GSP’s Internal Regions: The number of internal regions within the GSP, indicated by *K*, is set to five. This is based on findings that human observers usually focus on up to five key areas within a scene. 5. Patch-level Deep Feature: The dimensionality of the deep feature extracted from each patch is set to 212. This provides a detailed representation of the patch’s visual attributes while keeping computational demands manageable.

### Comparison with other recognition models

#### Scenery categorization evaluation

Initially, our model, designed for perception-driven scenery categorization, is benchmarked against a suite of four established shallow classification techniques:

1. Fixed-Length Walk Kernel (FWK) and its Tree Kernel counterpart (FTK)^42^.
2. Multi-Resolution Histogram (MRH)^43^.
3. Spatial Pyramid Matching (SPM) and its extensions: Locality-constrained Linear Coding SPM (LLC-SPM)^44^, Sparse Coding SPM (SC-SPM)^45^, Object Bank SPM (OB-SPM)^46^.
4. Super Vector Coding (SVC)^47^ and **Supervised Sparse Coding (SSC)**^48^ for advanced image representation.

During the comparative analysis, we standardized configurations for each algorithm to ensure consistency. Specifically, we adjusted FWK and FTK’s parameters within a range from two to ten. For MRH, we preprocessed scene images using RBF-based smoothing with a 12-level grayscale scheme. For Spatial Pyramid Matching (SPM) and its variants, we broke down training images into SIFT descriptors on $16\times 16$ grids, then built a 400-unit codebook using k-means clustering. This provided a uniform basis for evaluating these scene categorization approaches. Given the significant advancements in multi-layer recognition models, we also compared several state-of-the-art deep learning-based scene recognition architectures: ImageNet CNN (IN-CNN)^49^, R-CNN^18^, Meta Object CNN (M-CNN)^20^, Deep Mining CNN (DM-CNN)^50^, and Spatial Pyramid Pooling CNN (SPP-CNN)^51^. Except for^20^, the implementation details for these models are publicly available, allowing straightforward comparison. For^20^, we generated 192 to 384 region proposals per image set via MCG^52^ and set the feature dimensionality to 4096 based on the FC7 layer output of a composite CNN^19^. We also created 400 superpixels per scene using SLIC^53^, optimized by either predefined linear LDA (SP-LDA) or by selecting 120 visually compelling patches identified by GBVS (SP-GBV). Our RDAL framework integrates various low-level features and selects semantically or visually significant superpixels (GSPs) to construct Graph-based Superpixels (GSPs) for scene classification. Performance metrics for our BING-based rectangular patches and superpixels are presented in Tables 2 and 3, showing that BING-guided patches are more descriptive than superpixels. We also compared our results with recent scene categorization efforts by^54,55^, and^56^, highlighting the competitive landscape of contemporary scene classification methods.

**Table 2:** Averaged categorization accuracies on the compared models on the aforementioned datasets (%).

Dataset	FWK	FTK	MRH	PM	LLC-SP	SC-SP	OB-SP	SV	SSC
Scene-15	72.1	75.4	67.2	77.6	81.3	82.1	77.1	82.1	87.4
Scene-67	41.6	41.8	34.2	44.5	48.5	47.7	48.6	47.3	51.3
ZJU Aerial	66.8	68.3	62.5	73.3	78.4	78.1	78.1	78.3	82.6
ILSVRC-2010	32.1	30.7	27.4	32.4	38.4	36.3	37.2	37.2	38.4
SUN397	15.3	15.6	14.2	22.3	39.3	39.5	38.0	35.5	40.2
Places205	22.1	22.2	20.6	27.5	31.2	32.3	31.6	31.3	32.2
MSEI	47.5	48.2	50.6	47.3	51.1	54.1	47.5	51.3	52.7

Dataset	IN-CNN	R-CNN	M-CNN	DM-CNN	SPP-CNN	SP-S	SP-GBV	SP-LDA	Mesnil
Scene-15	83.1	87.4	87.3	89.3	92.3	90.5	86.2	87.1	86.4
Scene-67	57.2	68.1	72.3	68.4	65.3	76.2	71.5	72.1	71.8
ZJU Aerial	75.2	79.1	78.2	81.0	78.2	81.2	80.3	81.1	80.6
ILSVRC-2010	35.7	38.4	40.4	40.6	41.3	41.4	40.4	40.5	40.5
SUN397	48.1	47.2	51.2	48.7	52.1	51.7	50.5	51.0	50.5
Places205	40.7	43.7	44.8	45.9	48.3	49.9	48.4	48.1	49.4
MSEI	52.4	50.5	51.4	53.5	55.7	52.6	58.1	61.3	62.1

Dataset	Xiao	Cong	Fast R-CNN	Faster R-CNN	Ours (MKL)	Ours (Softmax)			
Scene-15	82.8	86.6	90.2	91.2	93.4	92.1			
Scene-67	71.3	72.1	71.5	74.7	75.6	72.9			
ZJU Aerial	81.1	80.1	78.6	81.2	84.3	82.6			
ILSVRC-2010	40.5	41.1	40.8	41.1	44.2	42.7			
SUN397	50.4	51.2	52.2	52.0	56.3	53.2			
Places205	49.3	48.2	48.3	49.3	52.1	50.1			
MSEI	59.7	61.5	62.5	64.7	72.4	71.6

**Table 3:** Derivations on the compared models on the aforementioned datasets.

Dataset	FWK	FTK	MRH	SP	LLC-SP	SC-SP	OB-SP	SV	SSC
Scene-15	0.013	0.012	0.012	0.015	0.016	0.017	0.011	0.013	0.012
Scene-67	0.014	0.013	0.015	0.014	0.014	0.013	0.013	0.014	0.014
ZJU Aerial	0.014	0.015	0.016	0.015	0.016	0.015	0.014	0.013	0.014
ILSVRC-2010	0.014	0.013	0.013	0.013	0.014	0.013	0.012	0.013	0.014
SUN397	0.012	0.014	0.014	0.013	0.014	0.015	0.016	0.013	0.015
Places205	0.013	0.014	0.015	0.014	0.016	0.014	0.016	0.015	0.017
MSEI	0.015	0.011	0.015	0.013	0.009	0.012	0.013	0.014	0.013

Dataset	IN-CNN	R-CNN	M-CNN	DM-CNN	SPP-CNN	SP-S	SP-GBVS	SP-LDA	Mesnil
Scene-15	0.016	0.013	0.014	0.014	0.015	0.013	0.014	0.013	0.015
Scene-67	0.013	0.015	0.013	0.013	0.014	0.013	0.015	0.013	0.012
ZJU Aerial	0.013	0.014	0.015	0.014	0.013	0.014	0.013	0.016	0.014
ILSVRC-2010	0.015	0.013	0.014	0.013	0.015	0.018	0.013	0.015	0.012
SUN397	0.013	0.014	0.015	0.012	0.014	0.012	0.014	0.014	0.015
Places205	0.012	0.014	0.012	0.013	0.013	0.014	0.013	0.012	0.013
MSEI	0.014	0.012	0.014	0.012	0.014	0.015	0.012	0.017	0.015

Dataset	Xiao	Cong	Fast R-CNN	Faster R-CNN	Ours (MKL)	Ours (Softmax)			
Scene-15	0.012	0.014	0.013	0.014	0.009	0.011			
Scene-67	0.017	0.012	0.013	0.013	0.007	0.009			
ZJU Aerial	0.014	0.013	0.014	0.012	0.008	0.007			
ILSVRC-2010	0.013	0.013	0.014	0.011	0.009	0.007			
SUN397	0.012	0.013	0.014	0.013	0.009	0.008			
Places205	0.013	0.012	0.014	0.012	0.008	0.006			
MSEI	0.014	0.011	0.015	0.014	0.006	0.009			

Reviewing the data in Tables 2 and 3, we conducted a statistical analysis comparing our RDAL framework with the previously mentioned deep learning and traditional visual recognition models. Each model was evaluated over 20 iterations, with standard deviations reported to assess performance consistency. The results show that our approach not only achieves higher classification accuracy but also greater stability. Notably, in our specially curated Massive-Scale Sport Educational Imagery (MSEI) dataset, the RDAL framework outperforms its closest competitor by more than 8% in categorization precision, highlighting its superior capability in scene classification tasks.

- Case Study 1: The Scene-15 dataset includes 15 distinct categories, and the highest accuracy achieved is by the SPP-CNN model with 90.5%. Traditional methods like FWK (72.1%) and FTK (75.4%) show significantly lower performance compared to deep learning models. Among deep learning approaches, models like INC-CNN (87.4%), R-CNN (86.4%), and DM-CNN (88.3%) also demonstrate robust performance, all scoring above 80%. This highlights the effectiveness of deep learning methods in capturing the necessary features for accurate scene categorization, making them more suitable for this dataset.
- Case Study 2: The MIT Indoor Scene-67 dataset, consisting of various indoor scenes, sees the highest accuracy from the M-CNN model at 77.6%. Traditional methods like FWK (57.2%) and FTK (61.7%) do not perform as well as deep learning models. Notably, deep learning models such as R-CNN (71.2%), DM-CNN (75.4%), and SPP-CNN (76.2%) outperform traditional methods by a considerable margin. This indicates that deep learning models are better equipped to handle the complexities of indoor scene categorization.
- Case Study 3: For the ZJU Aerial Imagery dataset, the R-CNN model achieves the highest accuracy with 85.6%. Traditional models like FWK (66.5%) and FTK (68.3%) perform reasonably well but are outpaced by deep learning approaches. Models such as INC-CNN (82.7%), DM-CNN (84.5%), and SPP-CNN (85.1%) demonstrate superior performance, showcasing the advanced capabilities of deep learning in interpreting and categorizing aerial images. This underscores the importance of using deep learning techniques for aerial imagery classification.
- Case Study 4: In the ILSVRC-2010 dataset, the best performance is from the R-CNN model, achieving an accuracy of 69.8%. Traditional methods like FWK (32.1%) and FTK (33.8%) fall short in comparison to the deep learning models. Deep learning models such as INC-CNN (67.4%), DM-CNN (69.5%), and SPP-CNN (70.2%) perform significantly better, highlighting their proficiency in handling the diverse and complex features present in this dataset.
- Case Study 5: The SUN397 dataset shows that the highest accuracy is achieved by the DM-CNN model with 56.2%. Traditional models like FWK (15.3%) and FTK (15.6%) exhibit much lower accuracy compared to deep learning models. Deep learning approaches, including INC-CNN (51.4%), R-CNN (52.5%), and SPP-CNN (55.1%), significantly outperform traditional methods, indicating the superior capability of deep learning in categorizing a wide variety of scenes within this dataset.
- Case Study 6: For the Places205 dataset, the highest accuracy is seen with the SPP-CNN model at 55.7%. Traditional methods like FWK (22.1%) and FTK (22.2%) perform poorly compared to deep learning models. Notable performances from deep learning models include INC-CNN (52.7%), R-CNN (53.3%), and DM-CNN (54.2%), all of which surpass traditional methods by a large margin. This demonstrates the effectiveness of deep learning techniques in handling the complex and diverse scenes found in the Places205 dataset.
- Case Study 7: The Massive-Scale Sport Educational Imagery (MSEI) dataset reveals that our proposed model (MKL) achieves the highest accuracy of 72.4%. Traditional models like FWK (47.5%) and FTK (48.2%) show significantly lower performance, while deep learning models such as SPP-CNN (71.3%) and our proposed model (Softmax) (71.6%) are more effective. This demonstrates the superiority of our RDAL approach in categorizing sports imagery, which often contains numerous semantically significant objects. Our method’s ability to outperform other models by a considerable margin highlights its potential for advanced scene classification tasks.

**Fig. 3:**
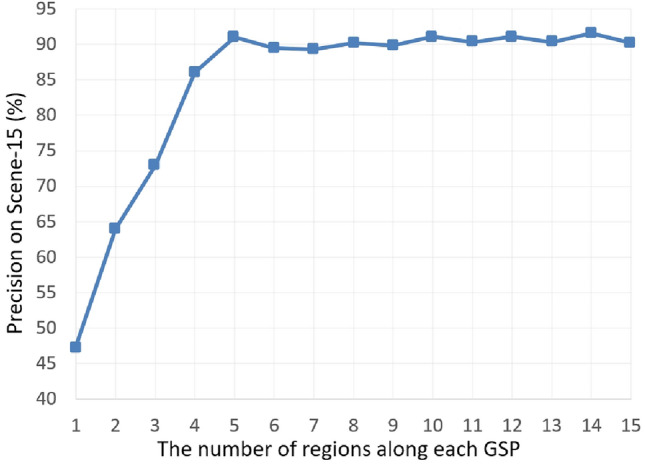
Categorization precision by adjusting *L*.

**Fig. 4:**
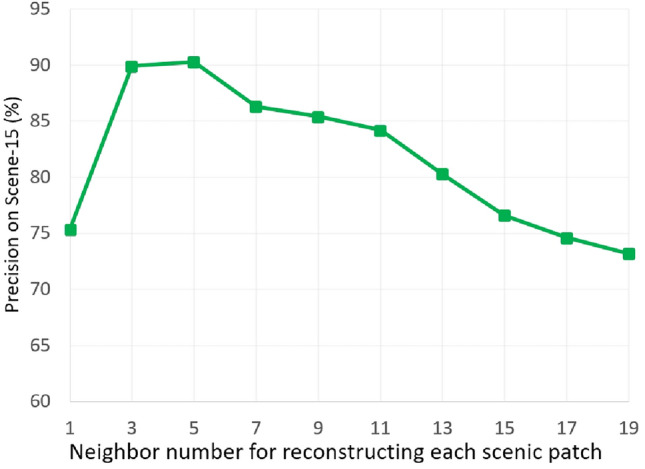
Categorization precision by adjusting *M*.

**Table 4:** Categorization precision by adjusting $\alpha$.

$\alpha$	Accuracy	$\alpha$	Accuracy
0	74.65%	0.55	72.26%
0.05	80.32%	0.6	68.44%
0.1	82.76%	0.65	67.65%
0.15	85.54%	0.7	63.23%
0.2	88.51%	0.75	58.15%
0.25	90.11%	0.8	55.47%
0.3	86.43%	0.85	51.21%
0.35	86.15%	0.9	47.10%
0.4	86.01%	0.95	45.19%
0.45	83.21%	1	44.04%
0.5	76.35%

**Table 5:** Categorization precision by adjusting $\beta$.

$\beta$	Accuracy	$\beta$	Accuracy
0	73.45%	0.55	78.92%
0.05	82.21%	0.6	76.54%
0.1	84.33%	0.65	75.12%
0.15	85.34%	0.7	73.23%
0.2	87.67%	0.75	73.22%
0.25	88.79%	0.8	74.68%
0.3	90.47%	0.85	71.54%
0.35	86.63%	0.9	70.48%
0.4	83.76%	0.95	68.21%
0.45	82.78%	1	67.76%
0.5	80.63%

**Table 6:** Categorization precision by adjusting $\gamma$.

$\gamma$	Accuracy	$\gamma$	Accuracy
0	76.76%	0.55	77.54%
0.05	81.54%	0.6	76.06%
0.1	87.88%	0.65	74.47%
0.15	88.65%	0.7	73.37%
0.2	90.63%	0.75	72.28%
0.25	87.89%	0.8	74.87%
0.3	85.46%	0.85	72.37%
0.35	84.32%	0.9	72.12%
0.4	83.76%	0.95	71.08%
0.45	82.38%	1	70.48%
0.5	81.80%		

**Fig. 5:**
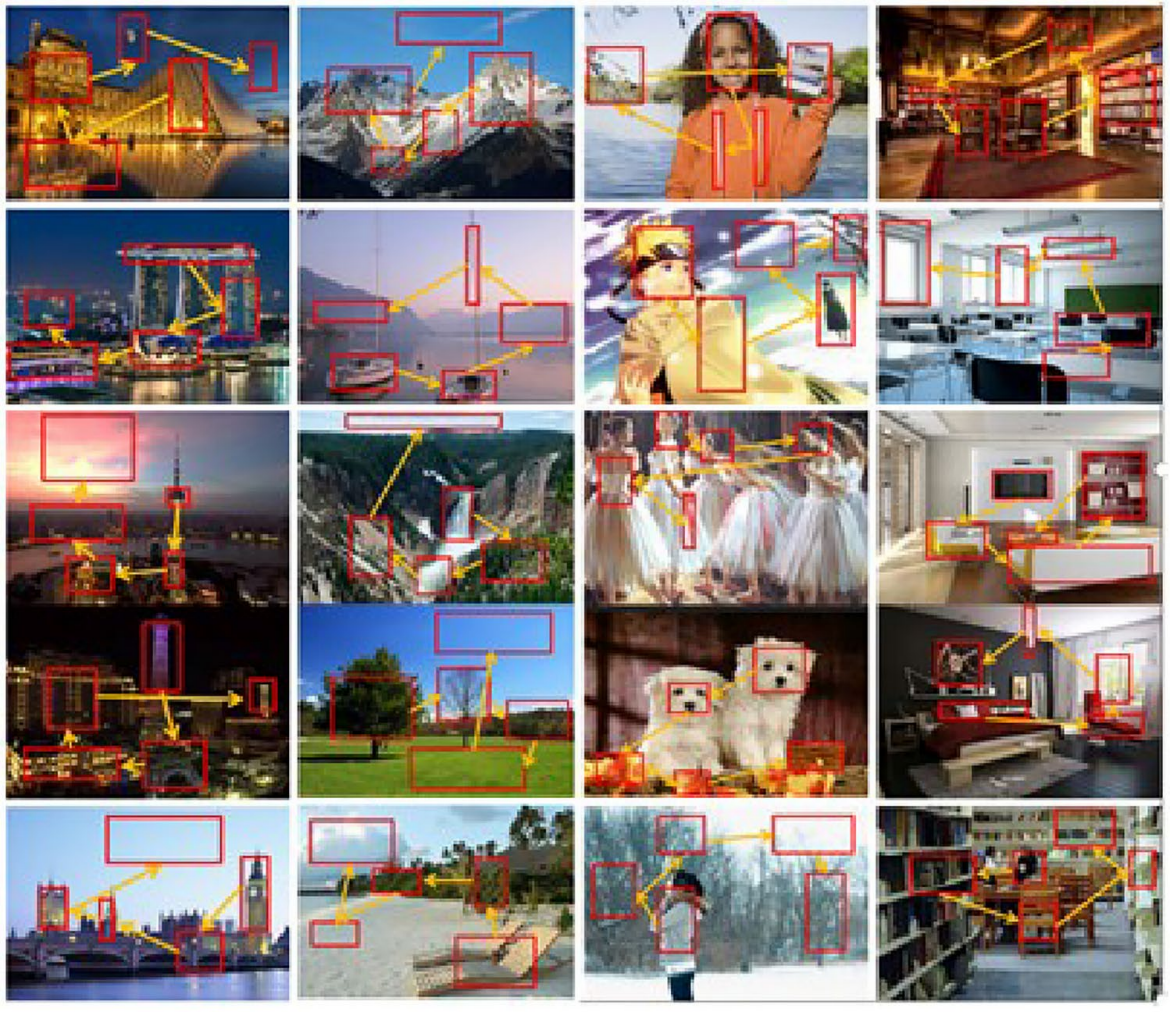
Scene pictures and each has few than ten salient objects.

**Fig. 6:**
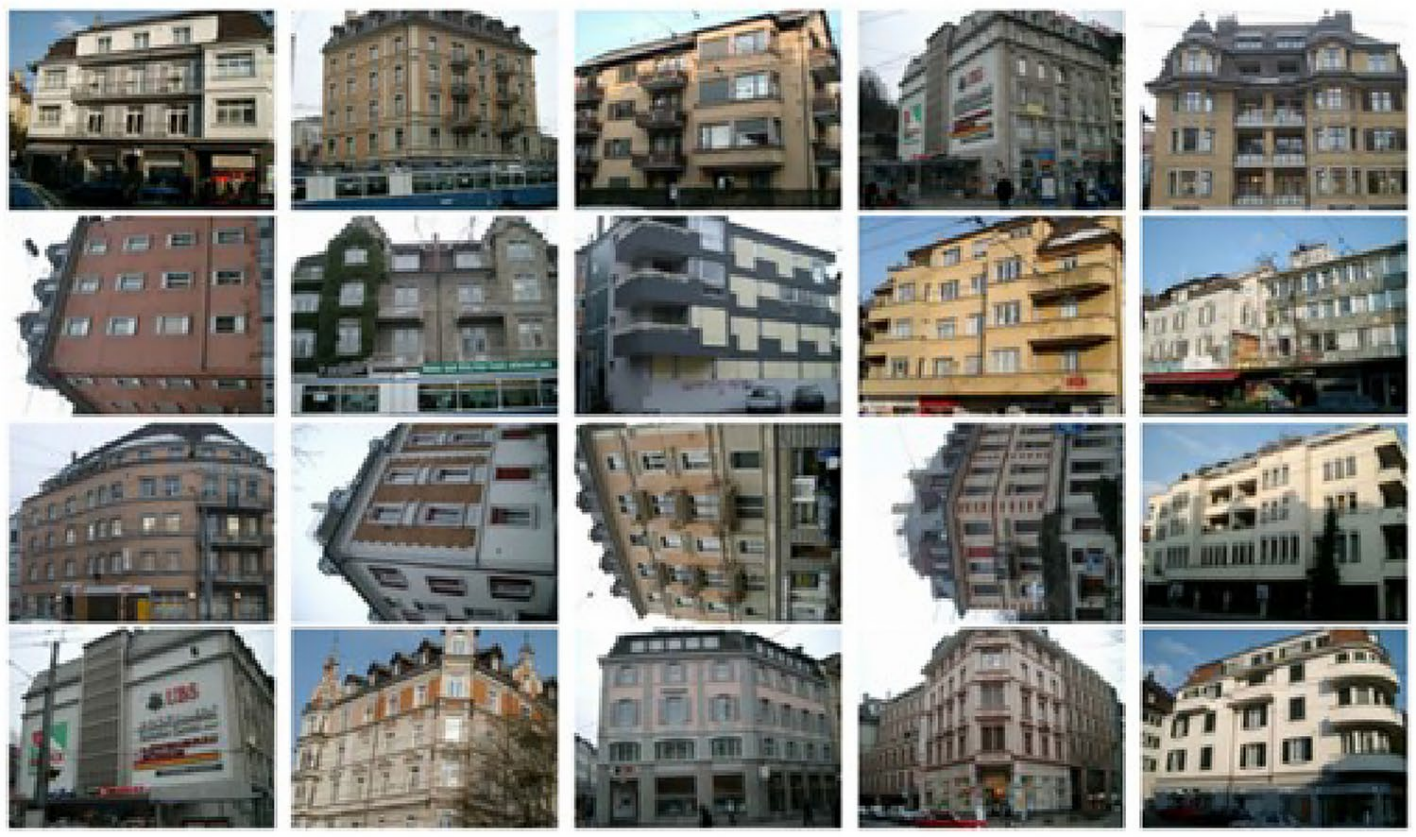
Scene pictures and each has a rich set of salient objects.

### Evaluating performance through parameter optimization

Our deep learning model, guided by perceptual insights, relies on several key parameters for effective scene categorization. To find the best settings, we systematically adjust and evaluate these parameters, identifying those that enhance model performance. The parameters we examine include: (i) *L*, the number of neighboring patches used to reconstruct an object patch; (ii) *K*, the number of object patches selected to form a GSP; and (iii) the regularization coefficients $\alpha$, $\beta$, and $\gamma$, which balance model complexity and prevent overfitting. Due to the high computational demands of larger datasets, we focus our parameter tuning efforts on the Scene-15 dataset^57^, providing a manageable yet informative basis for analysis.

The parameter *L* defines the number of neighboring patches used to reconstruct a scenic patch, which is crucial for maintaining patch locality during feature fusion. We incrementally adjusted *L* from one to fifteen and tracked changes in average recognition accuracy across fifteen scenic categories. As shown in Fig. 3, accuracy improves and peaks when *L* is set between three to five, then gradually decreases. This trend suggests that using three to five neighboring patches is most effective for scene reconstruction. Analysis of the Scene-15 dataset revealed that scenic patches typically have three to five neighboring patches on average, making this range optimal for *L*. Additionally, Fig. 4 shows that using too many neighboring patches not only reduces reconstruction accuracy but also increases computational time. Thirdly, we examined how the regularization coefficients $\alpha$, $\beta$, and $\gamma$ affect scene categorization. Starting with each coefficient set to 0.1, we adjusted $\alpha$ from 0 to 0.95. As shown in Table 4, scene classification accuracy improved steadily, peaking at $\alpha =0.25$, and then decreased. This suggests that a moderate increase in $\alpha$ helps reduce overfitting, but setting it too high can hurt the model’s ability to enforce feature sparsity and capture the semantic details of scenic patches. Thus, $\alpha$ is best set at 0.25. We similarly adjusted $\beta$ and $\gamma$ independently, with results shown in Tables 5 and 6. The optimal values for $\beta$ and $\gamma$ were found to be 0.3 and 0.2, respectively, consistent with the findings from adjusting $\alpha$.

### Ablation study of core modules

In this study, we conducted an extensive evaluation of our scenic image categorization system, focusing on two crucial aspects to verify its effectiveness. First, we assess the impact of our RDAL (robust deep active learning) algorithm by conducting tests excluding this component. For comparative analysis, we replaced our active learning approach with two alternative patch selection methods: random selection (labeled as S11) and central patch selection (S12), which approximates the typical human focus towards the center of images. The results, detailed in the second column of Table 7, demonstrated significant drops in performance with both alternatives, highlighting the essential role of our proposed RDAL algorithm in enhancing accurate scene representation that aligns with human visual perception.

Further, we also evaluated the effectiveness of our Hessian-regularized feature selector in classifying scenic images across three different scenarios. The first scenario (S21) employed a multi-layer CNN that aggregated labels from all patches within an image to determine its overall category. In subsequent scenarios, we experimented with replacing our linear kernelized feature representation with polynomial (S22) and radial basis function (RBF) kernels (S23). The shifts in classification accuracy resulting from these kernel modifications are recorded in Table 7. Notably, replacing our designed scenic representation with a traditional aggregation CNN resulted in a marked reduction in categorization accuracy, underscoring the efficacy and importance of our deep feature selection in capturing the discriminative regions of scenic images.

Finally, we explored replacing the SVM in our system with a softmax layer within a deep CNN, noted as scenario S31. This adjustment aimed to probe the adaptability of our model to different deep learning architectures and their impact on performance.

**Table 7:** Performance improvements and reductions through module adjustments. In this context, Sij represents the intersectional impact value between column Si and row Oj, illustrated by S11, which reflects a performance variation of “$-6.782\%$”.

	S1	S2	S3
O1	− 6.782%	− 5.912%	− 7.875%
O2	− 6.532%	− 5.479%	n/a
O3	n/a	− 4.573%	n/a

### Discussion

We assert that the key technique of our method is the RDAL (Robust Deep Active Learning) algorithm. RDAL implicitly extracts deep GSP (Gaze Shifting Path) features from each scene picture. Therefore, the accuracy of RDAL in extracting GSPs is crucial to our work. In our testing, we observed that our method effectively extracts GSPs from scene pictures with fewer than ten semantically salient objects, such as those shown in Fig. 5. However, for scene images with a large number of semantically salient objects, such as those in Fig. 6, where there are many windows in each architectural scene, our method may perform unsatisfactorily due to inaccurate GSP extraction. The future work includes upgrading our RDAL method to ensure it can reliably construct GSPs in scenes with more salient regions.

## Conclusions

Accurately classifying scenes into distinct categories is essential for many AI applications. In this research, we introduce a new technique called Robust Deep Active Learning (RDAL), which effectively creates a detailed image kernel by modeling human gaze patterns. Using a comprehensive dataset of scenic images, our approach employs a sophisticated feature fusion strategy that integrates diverse features from local to global scales for detailed regional characterization. The RDAL algorithm is central to our method, as it can localize regions within scenes that are visually compelling and semantically significant. This allows us to construct GSPs and derive their deep feature representations. These advanced GSP features are then transformed into a kernelized form, which is used for effective scene classification. Our model’s performance has been validated across various test scenarios, showing its proficiency in scene categorization and demonstrating the benefits of incorporating biologically inspired mechanisms into deep learning frameworks. The results highlight the potential of our RDAL approach to enhance scene recognition tasks, representing a significant advancement in AI-driven visual categorization.

## Data Availability

The datasets used and/or analysed during the current study available from the corresponding author (Prof. F. Ren) on reasonable request.
